# Type III interferon drives pathogenicity to *Staphylococcus aureus* via the airway epithelium

**DOI:** 10.1128/mbio.01130-24

**Published:** 2024-06-27

**Authors:** Silvia Pires, Katherine Kaiser, Dane Parker

**Affiliations:** 1 Department of Pathology, Immunology and Laboratory Medicine, Center for Immunity and Inflammation, Rutgers New Jersey Medical School, Newark, New Jersey, USA; St. Jude Children's Research Hospital, Memphis, Tennessee, USA

**Keywords:** *Staphylococcus aureus*, airway, type III interferon, interferons, lung

## Abstract

Type III interferon signaling contributes to the pathogenesis of the important human pathogen *Staphylococcus aureus* in the airway. Little is known of the cellular factors important in this response. Using *Ifnl2*-green fluorescent protein reporter mice combined with flow cytometry and cellular depletion strategies, we demonstrate that the alveolar macrophage is the primary producer of interferon lambda (IFN-λ) in response to *S. aureus* in the airway. Bone marrow chimeras showed reduced bacterial burden in IFN-λ receptor (IFNLR1)-deficient recipient mice, indicative that non-hematopoietic cells were important for pathogenesis, in addition to significant reductions in pulmonary inflammation. These observations were confirmed through the use of an airway epithelial-specific IFNLR knockout mouse. Our data suggest that upon entry to the airway, *S. aureus* activates alveolar macrophages to produce type III IFN that is subsequently sensed by the airway epithelium. Future steps will determine how signaling from the epithelium then exerts its influence on bacterial clearance. These results highlight the important, yet sometimes detrimental, role of type III IFN signaling during infection and the impact the airway epithelium plays during host–pathogen interactions.

**IMPORTANCE:**

The contribution of type III interferon signaling to the control of bacterial infections is largely unknown. We have previously demonstrated that it contributes to the pathogenesis of acute *Staphylococcus aureus* respiratory infection. In this report, we document the importance of two cell types that underpin this pathogenesis. We demonstrate that the alveolar macrophage is the cell that is responsible for the production of type III interferon and that this molecule is sensed by airway epithelial cells, which impacts both bacterial clearance and induction of inflammation. This work sheds light on the first two aspects of this important pathogenic cascade.

## OBSERVATION

Type III interferons (IFNs) signal via the JAK/STAT pathway after engagement of the type III IFN receptor (IFNLR), which is a heterodimer of interleukin-28 receptor (IL-28R) and IL-10RB that senses the ligands IFNλ1–3 (mice express λ2 and 3 only). A differentiating factor between type III IFN and the related type I IFN signaling pathway is the tissue distribution of the IFN-λ receptor, largely restricted to the epithelium and some immune subsets (1–7). The role of interferons in infectious disease varies depending upon the pathogen and body site and can be protective or inhibitory to pathogen clearance (5, 8, 9). Much of what is known on type III IFN signaling is from studies with viral pathogens. *Staphylococcus aureus* is an important human bacterial pathogen that is the major cause of pneumonia. We have previously demonstrated the importance of both type I and III IFNs in potentiating the pathogenesis of *S. aureus* infection in the airway (10–12), including the impact of type III IFN on the inflammasome. Much less is known on how the airway and its response to bacterial pathogens activates IFN-λ and then impacts the outcome to infection. Here, we report the identification of two major cellular contributors to this pathogenic cascade. We identify the contribution of alveolar macrophages to IFN-λ production and airway epithelial cells in recognition of IFN-λ to influence bacterial clearance and cytokine production.

**Fig 1:**
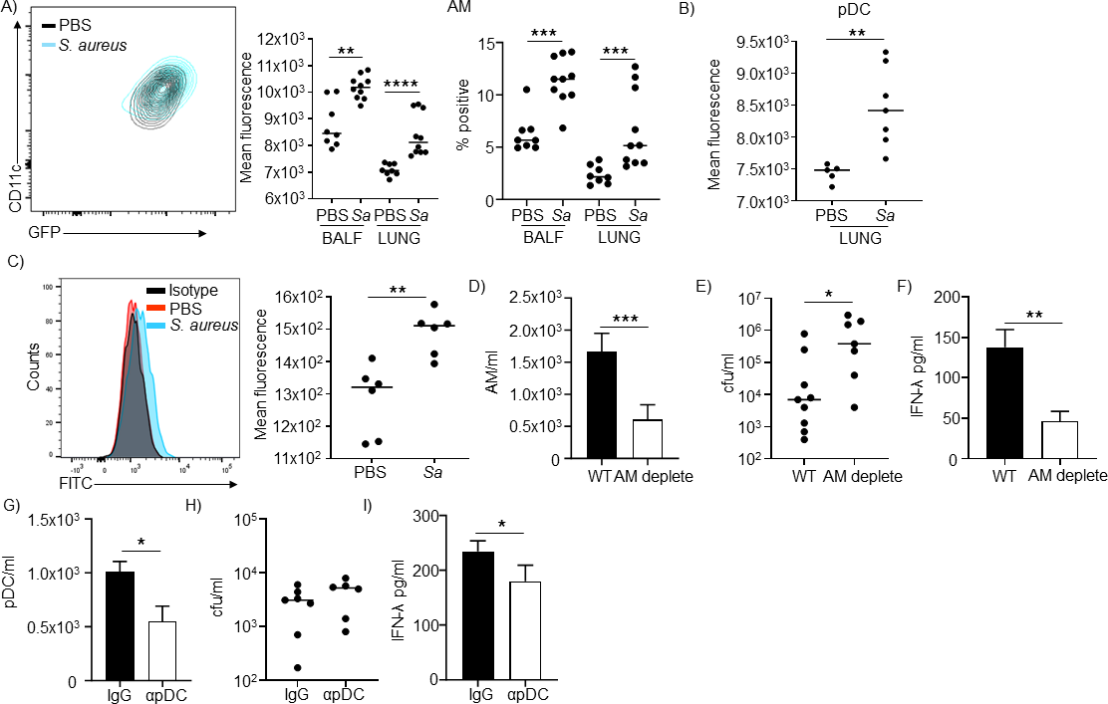
Alveolar macrophages are the major source of IFN-λ in response to *S. aureus* in the airway. *Ifnl2*-GFP mice were infected for 24 h with 5 × 10^7^ CFU of *S. aureus* USA300. BALF and digested lung cells were sorted by flow cytometry and examined for GFP production. (**A**) Gated on AM and (**B**) pDC in the lung. (**C**) C57BL/6 J mice were infected with *S. aureus,* and IFN-λ was detected in the lung using intracellular staining and flow cytometry. AM data are shown. Alveolar macrophages were depleted from C57Bl/6J mice using clodronate-loaded liposomes 24 h prior to infection with *S. aureus.* Mice were infected for 24 h. (**D**) Alveolar macrophage numbers in BALF. (**E**) Bacterial numbers in BALF of control and depleted animals. (**F**) IFN-λ was detected by ELISA in BALF of control and alveolar macrophage-depleted mice. WT *n* = 9, depleted *n* = 7. pDC were depleted through administration of αBST2 antibody or IgG control 24 h prior to infection with *S. aureus*. Mice were infected for 24 h. (**G**) pDC numbers in lung homogenates. WT *n* = 9, depleted *n* = 7. (**H**) Bacterial counts from BALF and (I) IFN-λ quantified by ELISA in BALF. WT *n* = 14, depleted *n* = 13. Each point represents a mouse. Lines display median. Data are from at least two independent experiments. Graphs display means and standard error. ****P < 0.0001, **P < 0.01, and *P < 0.05. *Sa, S. aureus*.

We first sought to determine the origin of IFN-λ in response to *S. aureus*. While a variety of cell types have been attributed to the source of IFN-λ, including epithelial cells, resident and plasmacytoid dendritic cells (pDC), lung-resident dendritic cells, and monocytes (6, 13–16), their roles can be pathogen specific and have not been assessed in the context of bacterial pathogens. We utilized a recently developed *Ifnl2*-green fluorescent protein (GFP) mouse to define the cell type(s) involved (17). GFP expression in response to *S. aureus* in different cell types revealed a shift in the alveolar macrophage (AM) population (Fig. 1A). AM had a significant increase in GFP, both from cells isolated from the bronchoalveolar lavage fluid (BALF) (*P* < 0.01) and from lung tissue (*P* < 0.0001), comparing *S. aureus*-infected mice to naïve controls (Fig. 1A). The only other detectable difference was a significant increase within the plasmacytoid dendritic cell (pDC) population of the lung (Fig. 1B; Fig. S1). To confirm our reporter mouse data, we conducted intracellular staining of IFN-λ in *S. aureus*-infected and naïve control mice. Intracellular staining was able to detect an increase in IFN-λ staining in the AM population but no other cell types (Fig. S2), indicating that the AM was likely to be the main source of type III IFN (Fig. 1C).

We conducted cellular depletion experiments to confirm our flow cytometry observations. AM were depleted from animals prior to infection using clodronate-loaded liposomes. Depletion of AM (Fig. 1D) led to an over eightfold increase in *S. aureus* (*P* < 0.05; Fig. 1E), highlighting the importance of AM in clearing *S. aureus* infection. Even with the significant increase in bacteria, the clodronate-treated animals still exhibited a 66% decrease (*P* < 0.01) in IFN-λ (Fig. 1F). While we did not observe a difference in IFN-λ induction in pDC from both of our flow cytometric experiments, we sought to further validate our AM data by depleting the pDC population. Depletion of pDC with αBST2 (Fig. 1G) did not alter the bacterial burden in the airways (Fig. 1H), with a small reduction in IFN-λ levels in the BALF (Fig. 1I). These data indicate that the AM is the primary IFN-λ source in response to *S. aureus* in the airway.

**Fig 2:**
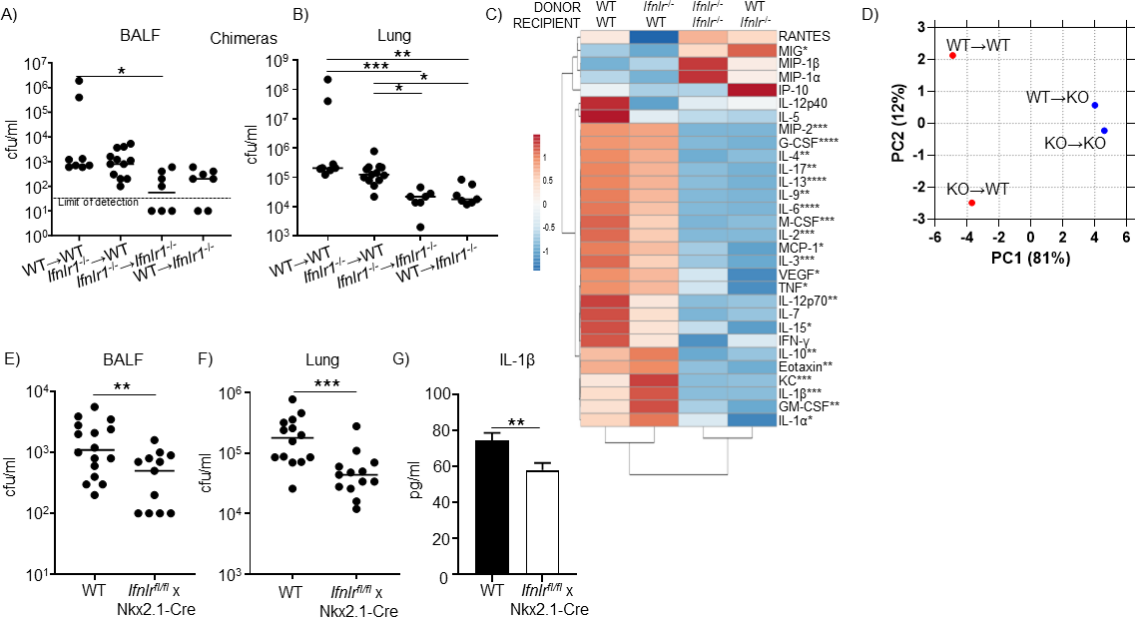
Type III IFN signaling from the epithelium influences clearance of *S. aureus*. Bacterial counts from (**A**) BALF and (**B**) lung of *S. aureus*-infected bone marrow chimeras using WT and *Ifnlr1*^−/−^ mice. Data assessed using a Kruskal–Wallis test with Dunn’s multiple comparisons. (**C**) Multiplex cytokine analysis of BALF from bone marrow chimeras. Heat map of fold changes showing average of group. WT-WT *n* = 9, knockout (KO)-WT *n* = 13, KO-KO *n* = 8, and WT-KO *n* = 8. Statistics comparing combined recipient groups calculated with a Student’s *t*-test. (**D**) Principal component analysis (PCA) of chimera BALF multiplex cytokine data. Nkx2.1Cre x *Ifnlr1-*LoxP mice were used to generate airway epithelial IFNLR knockout mice. Mice were intranasally infected with *S. aureus,* and bacteria were enumerated 24 h later in (**E**) BALF and (**F**) lung tissue. Data were compared using a Mann–Whitney test. (**G**) IL-1β ELISA in BALF. WT—*n* = 14, airway epithelial cell (AEC) KO—*n* = 12. Data were compared with a Student’s *t*-test. Each point represents a mouse; lines display median. Bars show mean with standard error. ****P < 0.0001, ***P < 0.001, **P < 0.01, and *P < 0.05.

Having now identified the AM to be the source of type III IFN following infection with *S. aureus*, we next set out to determine the cell responsive to this cytokine. No studies on bacteria in the airway have so far investigated the cell responsive to IFN-λ. Contrary to what is observed in the gut, we see type III IFNs playing a detrimental effect in *S. aureus* pathogenesis (10). We generated bone marrow chimeras using WT C57Bl/6J and *Ifnlr1*^−/−^ mice to first determine if the cells involved were hematopoietic, or not, in origin. We observed that recipient *Ifnlr1*^−/−^ mice, irrespective of receiving WT or *Ifnlr1*^−/−^ bone marrow had significantly less *S. aureus* than WT recipient mice. We detected >threefold less *S. aureus* in the BALF (*P* < 0.001; Fig. 2A) and >fivefold less in the lung tissue (*P* < 0.001; Fig. 2B), consistent with what we have observed in *Ifnlr1*^−/−^ mice (10), indicating a role for a non-hematopoietic-derived cell.

We have previously shown that in the absence of IFNLR1, there is a significant change in cytokine production in the airway. We quantified cytokines in BALF from our bone marrow chimera experiment using an inflammatory cytokine multiplex array. We observed significant reductions in many products that clustered based on the genetic background of the recipient mice (Fig. 2C). We did observe some increases in *Ifnlr1*^−/−^ recipient mice, with only CXCL9(MIG) being statistically significant. Principal component analysis (PCA) of the cytokine data further reflected that recipient expression of IFNLR1 was the largest driver of differential cytokine expression during *S. aureus* infection (Fig. 2D). The *Ifnlr1*^−/−^ recipient mice clustered together, disparate from WT recipient mice, with the first component accounting for 81% of the variance.

These data suggested the involvement of a non-hematopoietic cell. Given that IFNLR1 is predominantly expressed in the airway, we hypothesized the cell to be the airway epithelial cell (1, 2, 18). We generated airway epithelial cell (AEC)-specific Nkx2.1-cre *Ifnlr1^−/−^* knockout (KO) mice (Fig. S3) and infected them with *S. aureus*. Bacterial clearance was significantly improved in the AEC knockout (Fig. 2E&F), consistent with our bone marrow chimera studies and prior work (10). We have previously shown the connection between the inflammasome, IL-1β, and type III IFN signaling, and consistent with this, we observed a 22% (*P* < 0.01) decrease in IL-1β levels in the AEC knockouts (Fig. 2G). These data demonstrate that airway epithelial sensing of IFN-λ negatively impacts the host’s ability to clear infection.

Both type I and III interferons have varied roles depending upon the pathogen and infection site (2, 5, 18). In response to viral infection, the gut epithelium has been shown to be important by protecting the gut barrier in response to type III IFN (7, 19, 20). Recent studies have shown that expression of IFNLR1 in the airway epithelium can be detrimental to barrier protection during viral infection (13, 14). While not an acute bacterial infection, these reports are consistent with our own and other studies demonstrating the detrimental impact this pathway can have on bacterial pathogenesis in the lung (10, 11, 21–23).

To our knowledge, this is the first demonstration in the context of a bacterial pathogen that alveolar macrophages, a sentinel immune cell of the airway, are the primary producers of IFN-λ that is then recognized by the airway epithelium. We have also been able to demonstrate the importance of the epithelium sensing this cytokine and its contribution to pathogenesis during acute infection. While we have identified the first steps in this cellular response, it opens future avenues of investigation. Does the epithelium directly impact bacterial clearance through antimicrobial products or perturbation of the cytokine milieu, or alternatively, is it impacting function of the professional phagocytes or other immune responses in the airway? Understanding all these steps will inform host-directed approaches to better treat patients with this pathogen.

## SUPPLEMENTAL MATERIAL

Supplemental figures mbio.01130-24-s0001.pdf Figures S1 to S3.

Supplemental text mbio.01130-24-s0002.docx Methods.

Supplemental data mbio.01130-24-s0003.xlsx Original data.

ASM does not own the copyrights to Supplemental Material that may be linked to, or accessed through, an article. The authors have granted ASM a non-exclusive, world-wide license to publish the Supplemental Material files. Please contact the corresponding author directly for reuse.
