# Supplementary material for: Type III interferon drives pathogenicity to Staphylococcus aureus via the airway epithelium
Source: mBio. 2024 Jun 27;15(7):e01130-24. doi: 10.1128/mbio.01130-24 (PMC11253584; doi:10.1128/mbio.01130-24)
Supplement: Supplemental figures — Figures S1 to S3. [file mbio.01130-24-s0001.pdf]

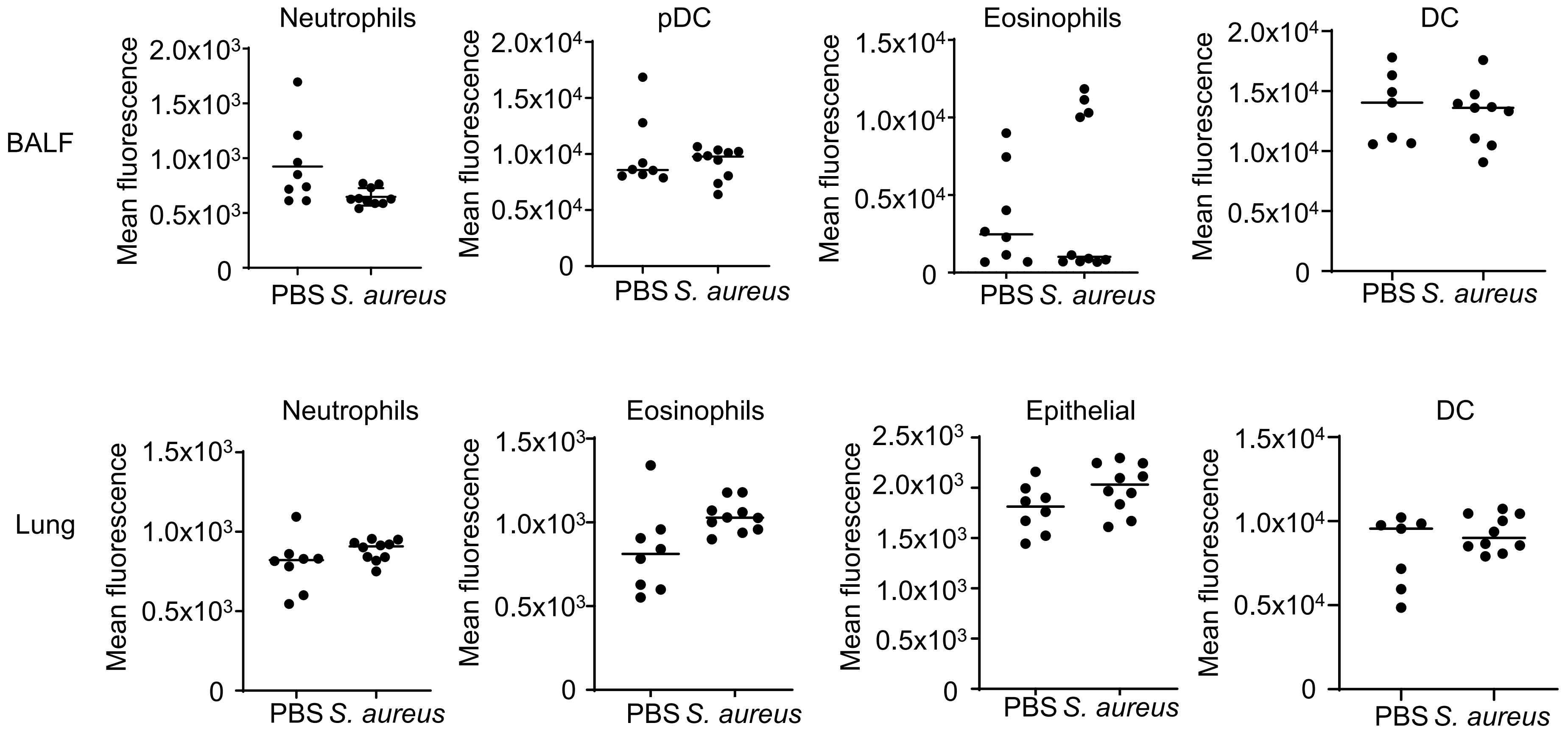

Supplemental Figure S1. IFNL-GFP expression data supplement to Figure 1A&B. *Ifnl2*-GFP mice were infected for 24 h with 5 x 10^7 cfu of *S. aureus* USA300. BALF and digested lung cells were sorted by flow cytometry and examined for GFP production. Lines display median. Each point represents a mouse.

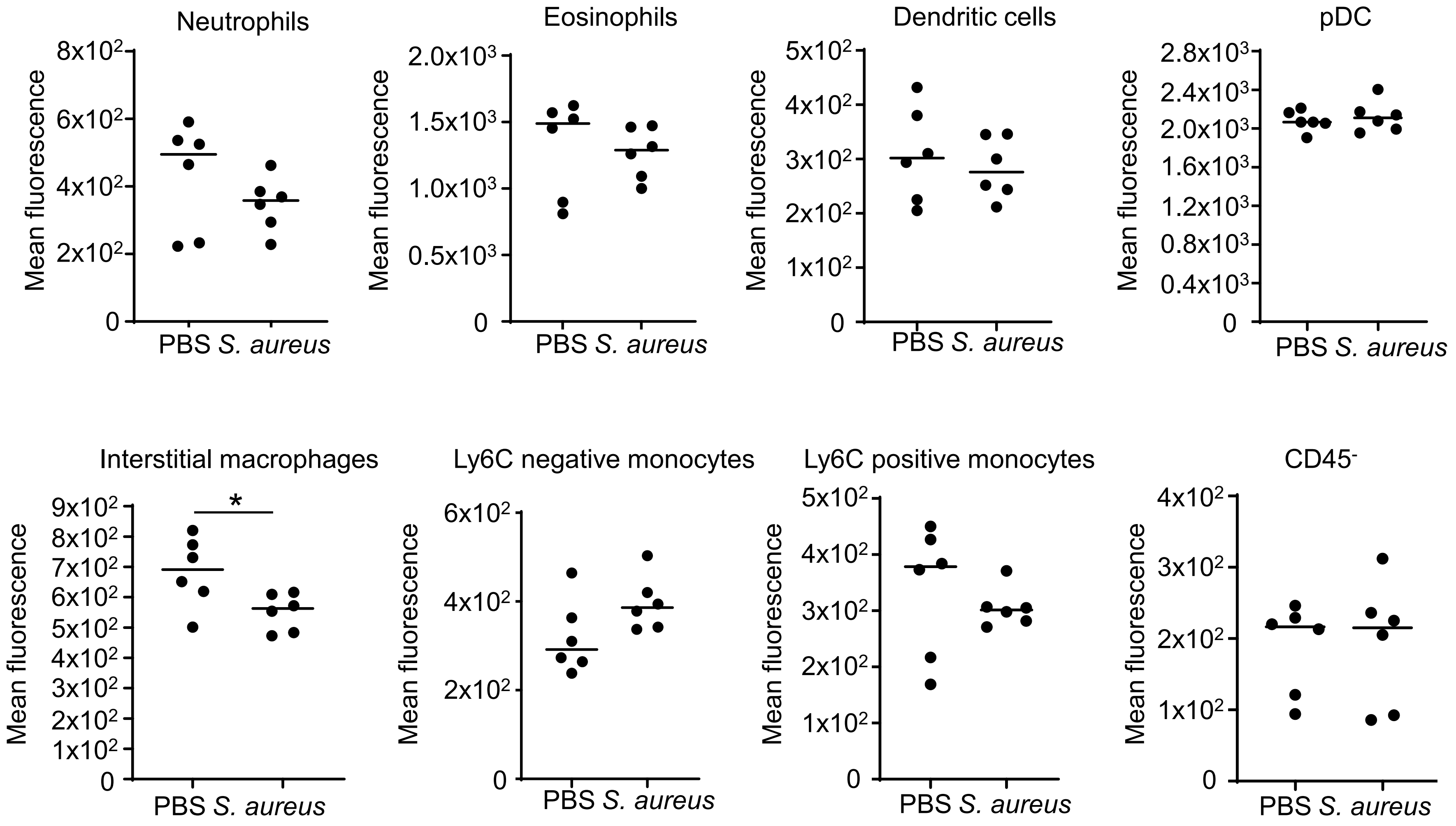

Supplemental Figure S2. Intracellular IFN-λ expression data to Figure 1C. C57BL/6J mice were infected with *S. aureus* and IFN-λ was detected in the lung using intracellular staining and flow cytometry. Lines display median. Each point represents a mouse. $*p<0.05$.

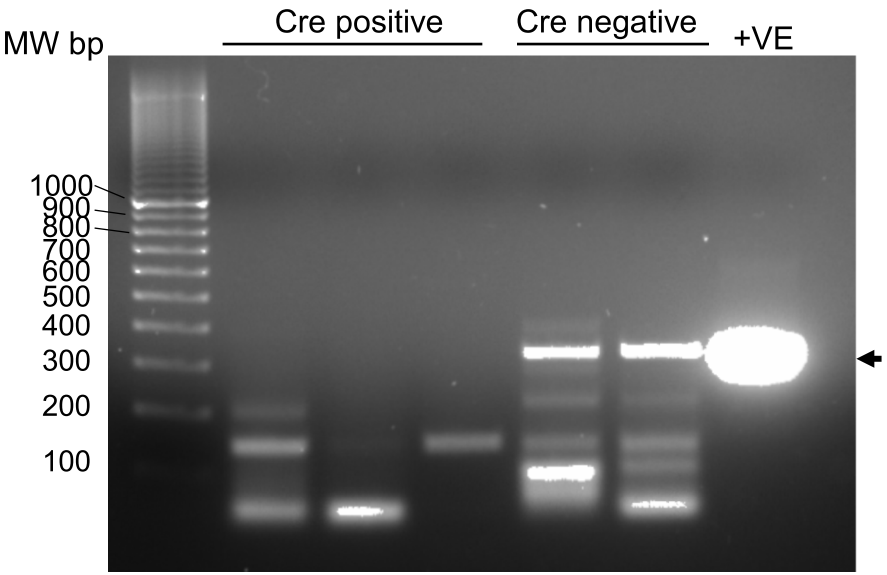

Supplemental Figure S3. RT-PCR gel electrophoresis of *Ifnlr*1. Airway epithelial cells were purified by cell sorting flow cytometry, RNA extracted and RT-PCR performed for the *Ifnrl1* gene. Control product showing expected size (+ve). Specific fragment is shown with arrow head. Other products are non-specific bands.
