# Supplementary material for: Type III interferon drives pathogenicity to Staphylococcus aureus via the airway epithelium
Source: mBio. 2024 Jun 27;15(7):e01130-24. doi: 10.1128/mbio.01130-24 (PMC11253584; doi:10.1128/mbio.01130-24)
Supplement: Supplemental text — Methods. [file mbio.01130-24-s0002.docx]

**Methods**

**Animal studies**

WT C57BL/6J, *Ifnlr1*^-/-^,  *Ifnlr1*^fl/fl^ (Lin *et al*, 2016), Nkx2.1-Cre [from Jackson Laboratories (Xu *et al*, 2008)] and *Ifnl2*-GFP mice (Sandoval *et al.*, 2021) were maintained in-house. Airway epithelial specific IFNLR mice were generated through mating *Ifnlr1*^fl/fl^ with Nkx2.1-Cre mice. Mice were genotyped using tail snips as before (Lin *et al.*, 2016). Detection of *Ifnlr1*  in airway epithelial cells was conducted with primers forward CACTGTTTACCTGACATGGCTTCCG (on exon 2) and reverse GCAGCTGGTAGGTAGCGTTGAC (on exon 4). Infection experiments involved intranasal delivery of 3 x 10^7^ cfu of *Staphylococcus aureus* FPR3757 as previously reported (Kim *et al*, 2023). Bacteria were enumerated using serial dilution from BALF and lung homogenate on chromogenic agar plates (BD Biosciences; *Staphylococcus aureus* Chromagar).

**Bone marrow chimeras**

Recipient mice received two doses of 600 rad 4 hours apart prior to receiving bone marrow intravenously. Bone marrow was isolated from the hind legs through homogenization prior to filtering and red blood cell lysis. Mice received 1 x 10^7^ cells in 100 μl intravenously. Mice received antibiotics in water (1 g/L ampicillin,1 g/L neomycin, 0,5 g/L vancomycin) for the first four weeks post irradiation. Experiments were conducted 3 months after transfer.

**Flow Cytometry**

*Ifn2*-GFP and WT were stained with combinations of fluorescently conjugated antibodies: CD45-AF700 (30-F11), Siglec-F-AF647 (E50-2440; BD Biosciences), Ly6C-PE-CF594 (AL-21; BD Biosciences), PCDA-1-BV510 (927), PCDA-1-PE (129c1), CD11c-BV605 (N418), Ly6G-PerCP/Cy5.5 (1A8), MHCII-APC-Cy7 (M5/114.15.2), and CD11b-PE-Cy7 (M1/70). IFN-λ production inside cells was detected using an IFN-λ2/3 antibody (R&D Systems; MAB17892) followed by a FITC-conjugated secondary (BioLegend). Antibodies were purchased from BioLegend unless otherwise stated. Viability was assessed using DAPI. Cells were acquired using a Fortessa X-20 cell analyzer (BD Biosciences). Analysis of flow cytometry data was performed using FlowJoV10 software. Cells in the airway were classified using previously described gating strategies (Pires *et al*, 2020) as follows: alveolar macrophages- CD45^+^Ly6C^-^SiglecF^+^CD11b^-^, neutrophils-CD45^+^Ly6C^+^CD11b^+^Ly6G^+^, interstitial macrophages-CD45^+^Ly6C^-^SiglecF^-^CD11b^+^CD11c^+^MHCII^+^, CD11b DC-CD45^+^Ly6C^+^CD11b^+^MHCII^+^CD11c^+^, Ly6C^+^ monocytes-CD45^+^Ly6C^+^CD11b^+^MHCII^-^Ly6G^-^, Ly6C^-^ monocytes-CD45^+^Ly6C^-^SiglecF^-^CD11b^+^CD11c^+^MHCII^-^, eosinophils-CD45^+^Ly6C^-^SiglecF^+^CD11b^+^, pDC-CD45^+^Ly6C^+^Cd11b^-^PDCA-1^+^ and epithelial cells-CD45^-^EpCAM^+^.

**Cytokine quantification**

IFN-γ was quantified in BALF using sandwich ELISA (R&D Systems duoset). Multiple cytokine concentrations in BALF were quantified by multiplex analysis (Mouse 32-plex; Eve Technologies). Cytokine heatmap was generated using ClustVis (Metsalu & Vilo, 2015), which also calculated loadings for PCA analysis.

**Cellular depletion studies**

Alveolar macrophages were depleted from mice using clodronate loaded or control liposomes (Martin *et al*, 2011; Pires *et al.*, 2020). Plasmacytoid dendritic cells were depleted through administration of 500 μg of anti-mouse CD317 (BST2, PDCA-1; BioXCell clone 927) or rat IgG2b κ control antibody 24 h prior to infection.

**Statistics**

Two samples were compared using an unpaired Student’s t test unless assumption of normality was violated (Shapiro Wilk test, p < 0.05), in which case Mann-Whitney tests were used. One-way analysis of variance (ANOVA) was used to assess multiple comparisons. A p-value <0.05 was considered significant. Statistical analyses were performed with the GraphPad Prism software version 9.0.1 for Windows (GraphPad, La Jolla, CA, USA). All experiments were conducted on at least two separate independent occasions.

**Ethics statement**

Animal work in this study was conducted in strict accordance with the recommendations in the Guide for the Care and Use of Laboratory Animals of the NIH (National Academies Press, 2011), the Animal Welfare Act, and US federal law. Protocols were approved by the Institutional Animal Care and Use Committee of Rutgers New Jersey Medical School of Newark, New Jersey, USA.

**Acknowledgements**

We thank Sergei Kotenko for the *Ifnlr1* floxed mouse and Joan Durbin for the *Ifnl2*-GFP mouse.

Numerical data for all figures is included as Data Supplement S1.
